# Effect of Synthetic Aβ Peptide Oligomers and Fluorinated Solvents on Kv1.3 Channel Properties and Membrane Conductance

**DOI:** 10.1371/journal.pone.0035090

**Published:** 2012-04-26

**Authors:** Maria I. Lioudyno, Matteo Broccio, Yuri Sokolov, Suhail Rasool, Jessica Wu, Michael T. Alkire, Virginia Liu, J. Ashot Kozak, Philip R. Dennison, Charles G. Glabe, Mathias Lösche, James E. Hall

**Affiliations:** 1 Department of Physiology and Biophysics, University of California Irvine, Irvine, Calfornia, United States of America; 2 Department of Physics, Carnegie Mellon University, Pittsburgh, Pennsylvania, United States of America; 3 Department of Molecular Biology and Biochemistry, University of California Irvine, Irvine, California, United States of America; 4 Department of Anesthesiology and Perioperative Care, University of California Irvine, Irvine, California, United States of America; 5 Department of Neuroscience, Cell biology, and Physiology, Wright State University, Dayton, Ohio, United States of America; 6 Department of Chemistry, University of California Irvine, Irvine, California, United States of America; 7 Center for Neutron Research, National Institute of Standards and Technology, Gaithersburg, Maryland, United States of America; 8 Department of Biomedical Engineering, Carnegie Mellon University, Pittsburgh, Pennsylvania, United States of America; University of Bristol, United Kingdom

## Abstract

The impact of synthetic amyloid β (1–42) (Aβ_1–42_) oligomers on biophysical properties of voltage-gated potassium channels Kv 1.3 and lipid bilayer membranes (BLMs) was quantified for protocols using hexafluoroisopropanol (HFIP) or sodium hydroxide (NaOH) as solvents prior to initiating the oligomer formation. Regardless of the solvent used Aβ_1–42_ samples contained oligomers that reacted with the conformation-specific antibodies A11 and OC and had similar size distributions as determined by dynamic light scattering. Patch-clamp recordings of the potassium currents showed that synthetic Aβ_1–42_ oligomers accelerate the activation and inactivation kinetics of Kv 1.3 current with no significant effect on current amplitude. In contrast to oligomeric samples, freshly prepared, presumably monomeric, Aβ_1–42_ solutions had no effect on Kv 1.3 channel properties. Aβ_1–42_ oligomers had no effect on the steady-state current (at −80 mV) recorded from Kv 1.3-expressing cells but increased the conductance of artificial BLMs in a dose-dependent fashion. Formation of amyloid channels, however, was not observed due to conditions of the experiments. To exclude the effects of HFIP (used to dissolve lyophilized Aβ_1–42_ peptide), and trifluoroacetic acid (TFA) (used during Aβ_1–42_ synthesis), we determined concentrations of these fluorinated compounds in the stock Aβ_1–42_ solutions by ^19^F NMR. After extensive evaporation, the concentration of HFIP in the 100× stock Aβ_1–42_ solutions was ∼1.7 μM. The concentration of residual TFA in the 70× stock Aβ_1–42_ solutions was ∼20 μM. Even at the stock concentrations neither HFIP nor TFA alone had any effect on potassium currents or BLMs. The Aβ_1–42_ oligomers prepared with HFIP as solvent, however, were more potent in the electrophysiological tests, suggesting that fluorinated compounds, such as HFIP or structurally-related inhalational anesthetics, may affect Aβ_1–42_ aggregation and potentially enhance ability of oligomers to modulate voltage-gated ion channels and biological membrane properties.

## Introduction

Complex mechanisms that may contribute to Alzheimer's disease (AD) involve genetic and environmental factors [1], [2] that under some, often unknown, conditions converge to initiate the onset of the neurodegeneration. Although the deposition of aggregated amyloid β (Aβ) peptide is the undisputed hallmark of the disease, it has been shown that Aβ also plays a physiological role in the brain in its non-aggregated state [3], [4], [5] and that it may function as an antibacterial peptide [6]. These diverse actions suggest that peptide conformation and aggregate size of Aβ oligomers, often characterized by their immunological properties, are crucial determinants of amyloid toxicity. Recent studies of *postmortem* brain samples demonstrated a significantly higher level of oligomers recognized by OC antibody [7] in AD patients compared to healthy controls [8]. These polyclonal antibodies recognize fibrillar oligomers that may represent fibril seeds or small pieces of fibrils. Importantly, the level of OC-stained fibrillar oligomers in the multiple brain regions correlates with the level of cognitive decline and other neuropathological hallmarks of Alzheimer's disease [8]. It is still unclear, however, which type of Aβ oligomer initiates neurotoxic reactions in the brain and what the molecular origins of these reactions are. *In vitro* studies suggest that small soluble Aβ oligomers, but neither monomeric nor fibrillar forms of the peptide, are neurotoxic [9], [10]. Multiple mechanisms by which oligomers cause calcium dysregulation, synaptic dysfunction, and ultimately neuronal cell death have been proposed. These include amyloid interactions with cellular membranes [11], [12], [13], the amyloid channel hypothesis [14], [15], amyloid effects on ion channels [16], [17], [18], [19], [20] and on neurotransmitter receptors [21], [22], [23], [24]. Results from artificial membrane models parallel those of cell toxicology and physiological studies [25], [26], in that small soluble oligomers, but not monomers or large aggregates such as fibrils, of amyloidogenic peptides and proteins affect the conductance [27], [28] and structural integrity [12] of lipid membranes. On the other hand, it has also been demonstrated that hexafluoroisopropanol (HFIP), frequently used as a solvent for the peptide in the preparation of amyloid oligomers, affects the conductance of bilayers and the ion flux across cell membranes [29]. Because Aβ has been associated with characteristic pathological changes, these findings are in the center of the debate whether the membrane effects of Aβ oligomers are at the core of AD etiology.

The important question of how the effects of endogenous, cell-derived Aβ peptide match those of synthetic peptide samples remains unresolved in large part because of the variety of methods for Aβ oligomer preparation used in different laboratories. In this work, we compare quantitatively the properties of Aβ oligomers formed by two common protocols, one using HFIP and the other NaOH as solvents to initiate the preparation of homogeneous oligomer samples. In a widely used procedure, HFIP is added to dissolve lyophilized Aβ peptide. Subsequently water is added to the monomeric peptide solution to initiate aggregation. To remove the fluorinated solvent the resulting solution is stirred in ambient air to allow the highly volatile HFIP to evaporate. The biophysical, toxicological and immunological characteristics of such preparations have been extensively studied [27], [30], [31], [32], and it was shown that they increase the conductance of lipid bilayers and influence both resident conductance mechanisms in cells and specific conductance mechanisms introduced into planar lipid bilayers [12], [28]. Yet it has also been demonstrated that similar effects are elicited by HFIP alone [29]. Another potential contaminant of the synthetic peptides is trifluoroacetic acid (TFA), which is commonly used in peptide synthesis and as solvent of lyophilized peptide. Although extensively removed during purification and lyophilization, it is toxic to cells at high concentration. Moreover, TFA activates some types of potassium channels [33]. It is therefore critical to determine which effects are due to Aβ oligomers and which to HFIP or TFA.

Here we use ^19^F NMR to quantify residual HFIP and TFA in synthetic Aβ oligomer samples, and test whether these compounds contribute to electrophysiological effects. We compare the effects of Aβ oligomers prepared with HFIP on voltage-gated Kv 1.3 potassium channels and on freestanding bilayer lipid membranes (BLMs) with those of HFIP-free oligomers prepared by initially dissolving the lyophilized peptide in NaOH. The oligomer preparations resulting from these protocols are further compared in their structural properties with dynamic light scattering (DLS) and characterized by their immunoreactivity.

## Results

### Size and conformation of Aβ_1–42_ aggregates prepared with HFIP or NaOH

**Figure 1:**
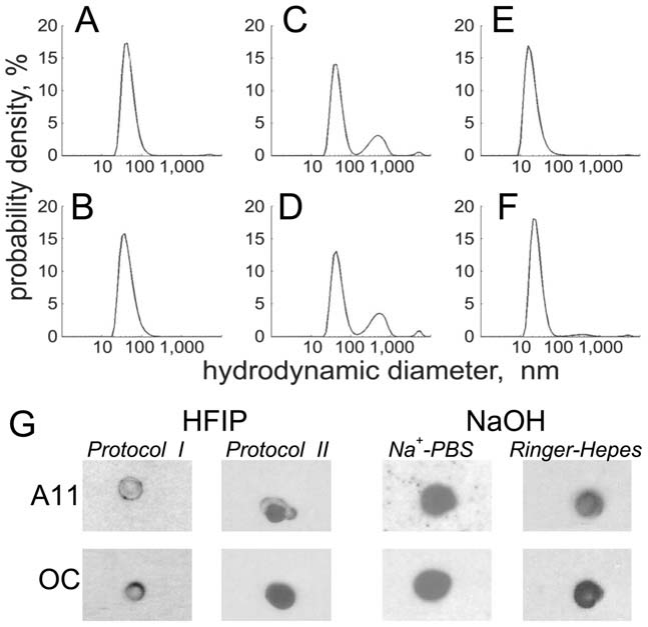
Size and conformation of Aβ_1–42_ aggregates. (A–F) Size distributions of Aβ_1–42_ aggregates at different time points, as determined with DLS. (A–D) Aβ_1–42_ samples prepared with HFIP: (A, B: *protocol* *I*; C, D: *protocol* *II*) (E, F) Aβ_1–42_ samples prepared with NaOH and incubated at pH = 7. A, C, F show samples incubated for 48 h and B, D, F show samples incubated for 4 d at room temperature. Data taken at *T* = 25±0.1°C. (G) Dot blots probed with A11 and OC antibodies show that both A11-positive and OC-positive material is contained in the Aβ_1–42_ samples after 4 days of incubation. Representative blots are shown (*n*≥2 for each condition).

To compare the sizes and size distributions of Aβ_1–42_ aggregates prepared by different protocols at similar time points after initiating oligomer formation, we used dynamic light scattering (DLS). Figure 1 shows distributions of Aβ_1–42_ aggregate sizes, evaluated under the assumption of spherical particles, obtained through inversion of the DLS data by a RILT algorithm and application of the Stokes-Einstein relation. Figure 1A–F show these distributions 2 and 4 days after initial solubilization with (Fig. 1A–D; *HFIP protocols* *I and II*) and without (Fig. 1E–F; *NaOH protocol*) HFIP. Both preparations are fairly stable in time but distinctly different, even under the uncertainty of the RILT inversion algorithm. The resulting distributions show main centroids that are shifted to slightly higher *d_h_* for those prepared with HFIP than those prepared with NaOH, *d_h_*≈40 nm, 41 nm, and 21 nm for *HFIP protocols* *I* and *II* and the *NaOH protocol*, respectively, at *t* = 48 h. In addition, the Aβ_1–42_ preparation from *HFIP protocol* *II* shows a greater proportion of larger aggregates in solution, as shown qualitatively by the secondary peak in Figs. 1C and D. The ratio of larger to smaller Aβ_1–42_ aggregates is ∼1/440 for *HFIP protocol II* and ∼1/4600 for the *NaOH protocol*. On the other hand, the main centroids of the size distributions do not shift significantly (from *d_h_*≈40 to 38 nm and from ≈41 to 43 nm for *HFIP protocols* *I* and *II*, and from *d_h_*≈21 to 23 nm for the *NaOH protocol*) between days 2 and 4, indicating stable aggregates persist throughout the typical time range of bilayer conductance and patch clamp experiments.

Not only the size but also the conformation of amyloid aggregates may affect their cytotoxicity and their ability to interact with cell membranes. Depending on the method and conditions of the Aβ sample preparation *in vitro*, different conformational states of aggregates may form, including prefibrillar oligomers, fibrillar oligomers, protofibrils, annular protofibrils, or mature fibrils [31], [34]. We used the conformation-specific antibodies, OC [7] and A11 [30], which recognize generic epitopes independent of amino acid sequence, to characterize Aβ samples. OC recognizes fibrillar oligomers and fibrils with a broad distribution of oligomer sizes from approximately 8 kDa to 200 kDa (∼3 nm to ∼8 nm). On the other hand, A11 recognizes prefibrillar oligomers of intermediate sizes. Figure 1G shows that, independently of the protocol used, the resulting Aβ_1–42_ solutions contained substantial amounts of both A11 and OC-positive oligomeric species previously implicated in AD pathology. Thus, qualitatively, all Aβ_1–42_ samples were similar, although they might contain different proportions of particular types of aggregates.

### Residual fluorinated solvents in Aβ_1–42_ samples

**Figure 2:**
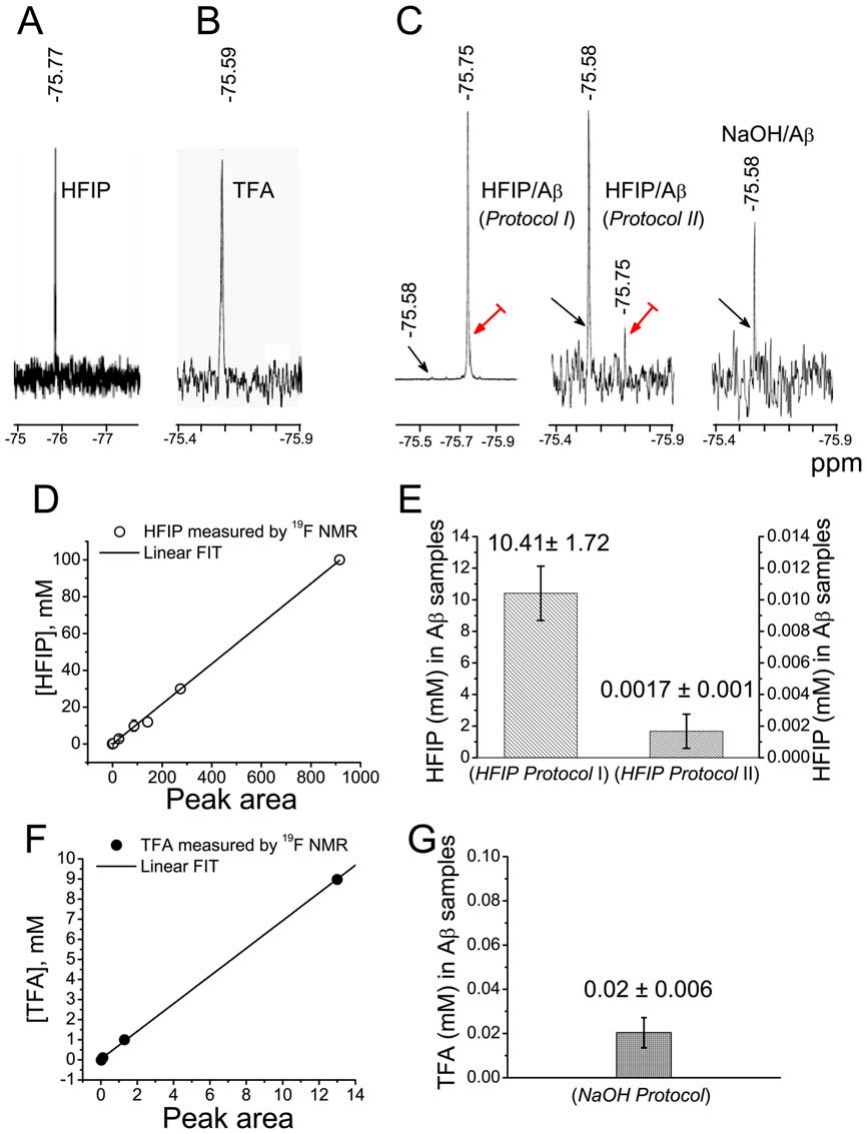
Quantification of HFIP and TFA in aqueous solutions by ^19^F NMR. (A) Aβ-free aqueous solution spiked with 0.1 mM HFIP. (B) Aβ-free aqueous solution spiked with 0.13 mM TFA. (C) ^19^F NMR spectra of Aβ_1–42_ oligomer samples prepared using *HFIP protocol* *I*, *HFIP protocol* *II*, and the *NaOH protocol*. The signal amplification differs greatly between spectra as indicated by the different noise levels. The concentrations of Aβ_1–42_ and HFIP *prior to evaporation* were 70 μM and 1.2 M. Black and red arrows indicate peaks originating from residual TFA and HFIP, respectively. (D, F) Calibration standards generated by integrating the area under the ^19^F peaks obtained from samples with known HFIP (D) or TFA (F) concentrations. The lines correspond to the best fits through the origin (*R* ^2^ = 0.999 for both fits). (E) HFIP concentrations ± S.E.M. in stock Aβ_1–42_ samples prepared according to *HFIP protocols* *I* (*n* = 6) and *II* (*n* = 7). (G) TFA concentration ± S.E.M. in stock Aβ_1–42_ samples prepared according to *NaOH protocol* (*n* = 2).

Because residual HFIP and/or TFA in Aβ_1–42_ samples may critically influence aggregate properties and contribute to effects observed in the electrophysiological experiments, we used ^19^F NMR as a sensitive method to quantify small concentrations of fluorinated substances in samples with or without Aβ_1–42_ (Fig. 2). The fluorine peak at about −75.7 ppm is characteristic of dissolved HFIP in the absence of peptide and originates from the two CF_3_ moieties (Fig. 2A). The peak at about −75.5 ppm is due to dissolved TFA in the absence of peptide (Fig. 2B). Aβ_1–42_ samples prepared with *HFIP protocol* *I*, for which we assumed in earlier work that HFIP was quantitatively removed [12], [27], [28], contain residual HFIP for which the signal is slightly shifted upfield to ≈−75.744 ppm (Fig. 2C, *left*). Even if the HFIP is evaporated from Aβ_1–42_ samples in open Eppendorf tubes (*HFIP protocol* *II*), a residual fluorine peak at –75.75 ppm was detected in ∼30% of the samples, albeit with a significantly reduced intensity (Fig. 2C, *middle*). In contrast, Aβ-free samples that initially contained HFIP, which was removed by extensive evaporation in open Eppendorf tubes, show no signal above noise at the spectral position characteristic of HFIP (data not shown). As expected, this signal was also not observed in samples prepared by using NaOH as solvent (Fig. 2C, *right*), whereas TFA peaks (at –75.5 ppm) were detected in all Aβ_1–42_ oligomer samples regardless of the preparation protocol used.

Calibration curves were determined for the HFIP and TFA concentrations in aqueous solutions by using samples with known concentrations of the fluorinated compounds and determining the signal strengths at standardized instrument settings (Fig. 2D, F). In Aβ_1–42_ stock solutions prepared by *HFIP protocols I* ([Aβ_1–42_] = 70 μM) and *II* ([Aβ_1–42_] = 100 μM), the average contents of HFIP estimated from the observed peak areas in the ^19^F NMR signal were 10.41±1.72 mM and 1.7±1 μM, respectively (Fig. 2E). After dilution of these stock solutions to the Aβ_1–42_ concentration used for electrophysiological experiments ([Aβ] = 1 to 6 µM), the HFIP concentration would be <1 mM for samples prepared following *protocol I*, and not more than 0.1 µM, for samples prepared following *protocol* *II*. The concentration of residual TFA in 70 µM Aβ_1–42_ stock solutions was 20 µM (Fig. 2G).

### HFIP and TFA affect neither Kv1.3 channels nor BLM conductance

A recent study showed that HFIP alone modulates the properties of biological membranes [29]. In the light of the hypothesis that Aβ oligomers affect neuronal membranes and may interfere with cognitive functions by a membrane-dependent mechanism, this raises the question if effects reported earlier as Aβ oligomer-specific are augmented by or entirely attributable to the presence of residual HFIP. Furthermore, TFA can activate ATP-sensitive potassium channels at concentrations as low as 0.05 mM [33].

**Figure 3:**
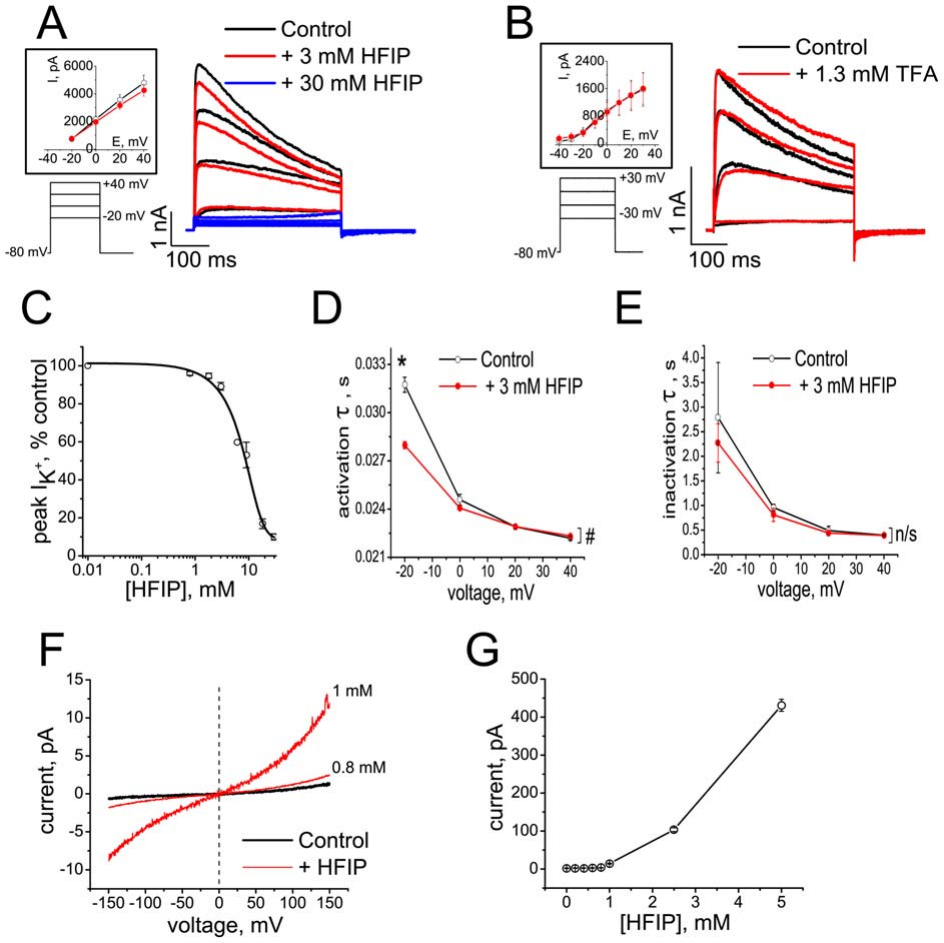
HFIP and TFA effects on Kv1.3 channel currents and BLM conductance. (A) Representative current traces before (black) and after (red and blue) application of HFIP (*n* = 5 cells). (B) Representative current traces before (black) and after (red) application of TFA (*n* = 3 cells). The current-voltage relations before and after application of HFIP (A) or TFA (B) are shown in the inserts. (C) Dose-response curve for the HFIP effect on peak K^+^ currents, evoked by depolarizing steps to +40 mV. IC_50_ = 5.2±3.4 mM from fitting the data (mean ± S.E.M., *n* = 5 cells for each concentration) to a Boltzmann function. (D) Activation of Kv 1.3 current is accelerated by HFIP (Data shown as mean ± S.E.M., *n* = 3 cells). The effect of HFIP on the activation time constant was significant (F = 50.6; ^#^P = 0.01, Two-way RM-ANOVA) with significant interaction between FactorA (treatment) and FactorB (voltage) (F = 155), and by Pairwise Comparisons at −20 mV (*P = 7.9969×10^−6^, Tukey test). (E) The inactivation kinetics of Kv 1.3 current are not significantly affected by HFIP (mean ± S.E.M., *n* = 3 cells, ^#^P = 0.36369, Two-way RM-ANOVA). (F) Representative *I*/*V* curves recorded on DOPC/DOPE BLMs in the presence of HFIP. (G) Dose-dependence of HFIP-induced currents through BLMs at +150 mV (mean ± S.E.M. for *n* = 7).

Therefore, we first quantify the effects of HFIP and TFA on Kv 1.3 currents and BLM conductance. In patch clamp experiments, high concentrations of HFIP (3–30 mM) reduce the Kv 1.3 peak current amplitude (Fig. 3A, C). In addition, HFIP accelerates kinetics of the K^+^ current at lower concentrations (≈1–3 mM), although the effect on inactivation time constants is not significant (Fig. 3D, E). TFA had no effect on Kv 1.3 currents at concentrations up to 1 mM and only at higher concentrations slowed the inactivation rate, consistent with the low-pH effect on C-type inactivation [35] (Fig. 3B). Figure 3F, G shows that HFIP itself also increases the BLM conductance in a dose-dependent manner, starting at concentrations >1 mM. A characteristic threshold for HFIP effects on K^+^ currents through Kv1.3 as well as on BLM conductance is ≈1 mM. While this threshold is comparable with the HFIP level in Aβ_1–42_ samples prepared by *HFIP protocol* *I*, it is significantly higher than the HFIP level in Aβ_1–42_ samples prepared by *HFIP protocol* *II*. Thus neither HFIP nor TFA are present in Aβ oligomer samples prepared by *protocol* *II* or *NaOH protocol* at the concentrations that could contribute to the observed effects on K^+^ currents or BLM conductances.

### Aβ_1–42_ oligomers prepared by *HFIP protocol I*: Effects on Kv 1.3 currents and BLM conductances

Aβ_1–42_ solutions prepared by *HFIP protocol* *I* contain heterogeneous populations of aggregates in which different oligomeric species are present at distinct time points of the incubation period. However, DLS shows that Aβ_1–42_ oligomers do not significantly change between days 2 and 4 after initiation of the preparation, a time range in which the electrophysiological measurements were conducted.

**Figure 4:**
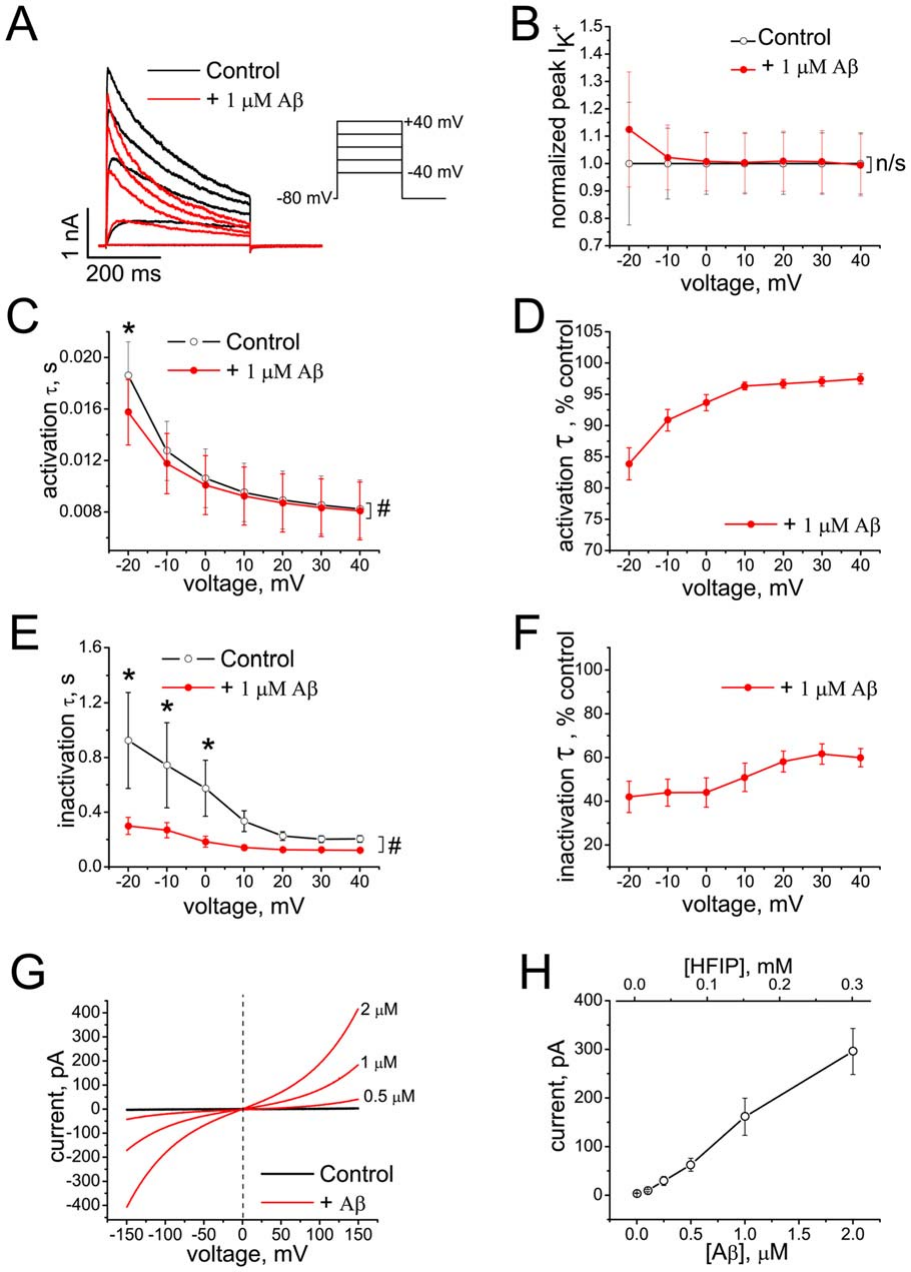
Effect of Aβ_1–42_ oligomers (*HFIP protocol* *I*) on Kv 1.3 currents and on BLM conductance. (A) Representative K^+^ currents evoked by depolarizing voltage steps from holding potential of −80 mV before (black) and after (red) application of Aβ_1–42_ oligomers. (B) Peak K^+^ currents normalized to mean control values at different voltages before (black) and after application of Aβ_1–42_ oligomers (red). Data are shown as mean ± S.E.M. (*n* = 6 cells). HFIP had no significant (n/s) effect on the peak current (F = 0.17; P = 0.69, Two-way RM-ANOVA). (C–F) Activation and inactivation kinetics of K^+^ currents before (black) and after application of Aβ_1–42_ oligomers (red) shown in absolute (C, E) and normalized (D, F) values of time constants at different voltages (mean ± S.E.M., *n* = 6 cells). The effect of Aβ on the activation time constant was significant in Tests of Within-Subjects Effects (F = 46.8; ^#^P = 4.7×10^−4^, Two-way RM-ANOVA), with significant interaction between FactorA (treatment) and FactorB (voltage) (F = 25.9), and by Pairwise Comparisons at −20 mV (*P = 2.08×10^−4^, Tukey test). The effect of Aβ on the inactivation time constant was also significant in Tests of Within-Subjects Effects (F = 8.1; ^#^P = 0.04, Two-way RM-ANOVA), and by Pairwise Comparisons at −20 mV, −10 and 0 mV (*P<0.05; Tukey test). (G) Representative *I*/*V* curves recorded on DOPC/DOPE BLMs before (black) and after (red) application of Aβ_1–42_ oligomers. (H) Dose-dependence of Aβ_1–42_-induced currents at +150 mV across BLMs (mean ± S.E.M., *n* = 11 experiments, out of a total of 16, in which the effect was observed). HFIP concentrations estimated from ^19^F NMR spectra of the Aβ_1–42_ stock solutions are shown on the top axis.

The effect of Aβ_1–42_ aggregates prepared according to *HFIP protocol* *I* on the currents and kinetics of Kv 1.3 is shown in Fig. 4. The K^+^ current is strongly affected by acute perfusion of the cells. The peak current amplitude tends to increase at low voltages and to decrease at the higher voltages (Fig. 4 A, B), and both activation and inactivation kinetics are accelerated in a voltage-dependent manner (Fig. 4 C–F). The concentrations of HFIP in the extracellular solutions were less than 1 mM, a concentration too small to account for the observed effects by the action of HFIP alone, as a comparison of Figs. 3 and 4 shows.

Although Aβ_1–42_ had no effect on ionic currents at the holding membrane potential (−80 mV) recorded in cells, the conductance of the artificial lipid bilayer membranes was increased by Aβ_1–42_ aggregates in a dose-dependent manner, consistent with our earlier finding [28]. Figure 4G shows representative current-voltage curves measured in dioleoylphosphatidylcholine/dioleoylphosphatidylethanolamine (DOPC/DOPE) BLMs bathed on both sides with 10 mM KCl. Panel H shows the average current at +150 mV induced in such bilayers as a function of Aβ_1–42_ concentration. In these experiments, the residual HFIP concentration (Fig. 4H) is ≤0.3 mM, as estimated by ^19^F NMR, and a comparison of Figs. 3F, G and 4G, H reveals that the effects of the peptide at 2 µM Aβ_1–42_ are still significantly greater than those of 1 mM HFIP alone.

### Aβ_1–42_ oligomers prepared by *HFIP protocol* *II*: Effects on Kv 1.3 currents and BLM conductances

Aβ_1–42_ samples prepared by *HFIP protocol* *II* contain a population of larger aggregates in addition to smaller aggregates contained in both *HFIP protocol* *I-*, and *II-* samples as indicated by the secondary peak in Figs. 1C and D.

**Figure 5:**
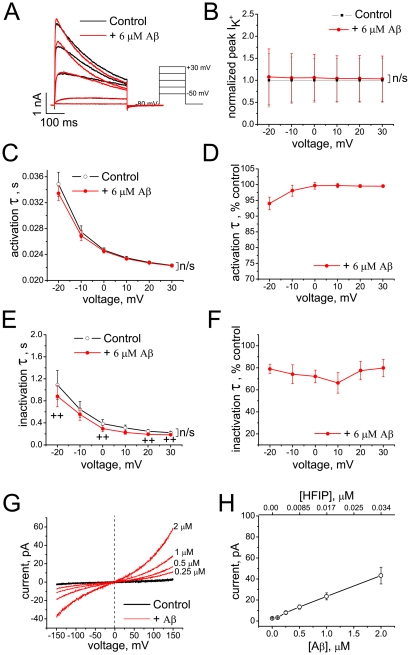
Effects of Aβ_1–42_ oligomers (*HFIP protocol* *II*) on Kv 1.3 currents and on BLM conductance. (A) Representative K^+^ currents evoked by depolarizing voltage steps from the holding potential of −80 mV before (black) and after (red) application of Aβ_1–42_ oligomers. (B) Peak K^+^ currents normalized to mean control values at different voltages after application of Aβ_1–42_ oligomers. The differences in peak current amplitude before and after application of Aβ are not significant (F = 3.9; P = 0.08 two-way RM-ANOVA). (C–F) Activation and inactivation kinetics of K^+^ currents before (black) and after application of Aβ_1–42_ oligomers (red) shown in absolute (C, E) and normalized (D, F) values of time constants at different voltages (mean ± S.E.M., *n* = 4 cells). The effect of Aβ on the activation time constant was not significant in Tests of Within-Subjects Effects (F = 4.4; ^#^P = 0.07, Two-way RM-ANOVA). ANOVA analysis also revealed no significant effect of Aβ on the inactivation time constant (F = 8.7; ^#^P = 0.05, Two-way RM-ANOVA), however two-way paired t-Test, (^++^P<0.05) showed significant differences between mean time constant measured before and after treatment with Aβ, revealing the trend. (G) Representative *I*/*V* curves recorded on DOPC/DOPE BLMs before (black) and after (red) application of Aβ_1–42_ oligomers. (H) Dose-dependence of Aβ-induced currents at +150 mV across BLMs (mean ± S.E.M., *n* = 7 experiments, out of a total of 9, in which the effect was observed).

The *HFIP protocol* *II* samples tended to modulate Kv 1.3 potassium current properties (Fig. 5A–F) and increased bilayer conductance (Fig. 5G, H) in a similar fashion to Aβ_1–42_ samples prepared by *HFIP protocol* *I*, although the effects were only observed at higher concentrations of Aβ_1–42_. At the lower concentrations (1 and 3 µM) Aβ_1–42_ produced no effect on potassium currents (data not shown). Current amplitudes and the activation kinetics were not significantly affected (Fig. 5A, B, and C, D) and the effects on inactivation kinetics were less prominent (Fig. 5E, F), reaching significance level when analyzed by paired t-Test, but not by repeated measures ANOVA. These effects can not be attributed to residual free HFIP in the solution, as the fluorine peaks revealed by ^19^F NMR in Aβ_1–42_ samples prepared by *HFIP protocol* *II* are extremely small.

### Aβ_1–42_ oligomers prepared by the *NaOH protocol*: Effects on Kv 1.3 currents and BLM conductances

Although Aβ_1–42_ samples prepared by the *NaOH protocol* contain a larger aggregates, in addition to smaller aggregates population, the proportion of larger aggregates in Aβ_1–42_ samples prepared by the *NaOH protocol* is much lower than in the Aβ_1–42_ samples prepared by *HFIP protocol* *II* (Figs. 1C–F).

**Figure 6:**
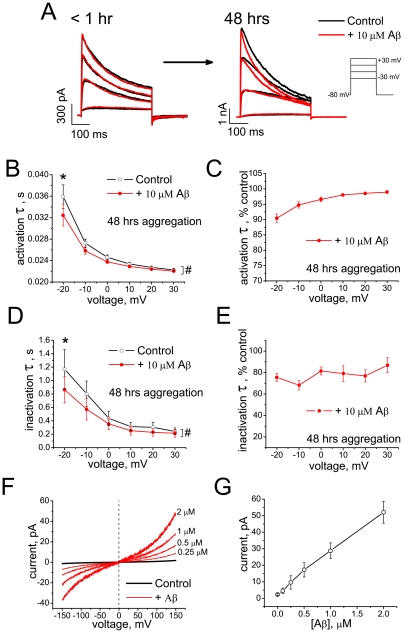
Effect of HFIP-free Aβ_1–42_ oligomers (*NaOH protocol*) on Kv 1.3 current and on BLM conductance. (A) Representative K^+^ currents evoked by depolarizing voltage steps from the holding potential of −80 mV before (black) and after (red) Aβ_1–42_ application. Note, that Aβ_1–42_ samples aggregated for less than 1 hr and presumably contained monomeric peptide, had no effect on K^+^ current (A, *left*), whereas samples aggregated for 48 hrs produced characteristic effect on K^+^ current kinetics (A, *right*). (B–E) Activation and inactivation kinetics of K^+^ currents before (black) and after application of Aβ_1–42_ oligomers (red) shown in absolute (B, D) and normalized (C, E) values of time constants at different voltages (mean ± S.E.M., *n* = 4 cells). The effect of Aβ on the activation time constant was significant in Tests of Within-Subjects Effects (F = 34.5; ^#^P = 0.009, Two-way RM-ANOVA) with significant interaction between FactorA (treatment) and FactorB (voltage) (F = 38.03), and by Pairwise Comparisons at −20 mV (*P = 0.006, Tukey test). The effect of Aβ on the inactivation time constant was also significant in Tests of Within-Subjects Effects (F = 19.1; ^#^P = 0.022, Two-way RM-ANOVA) with significant interaction between FactorA (treatment) and FactorB (voltage) (F = 7.1), and by Pairwise Comparisons at −20 mV (*P = 0.016, Tukey test). (F) Representative *I*/*V* curves recorded on DOPC/DOPE BLMs before (black) and after (red) application of Aβ_1–42_ oligomers. (G) Dose-dependence of Aβ-induced currents at +150 mV across BLMs (mean ± S.E.M., *n* = 5 experiments, out of a total of 12, in which the effect was observed).

HFIP-free Aβ_1–42_ oligomer samples prepared by the *NaOH protocol* modulated the Kv 1.3 current similarly to Aβ oligomers prepared by *HFIP protocol I* (Fig. 6A–E). However, higher peptide concentrations (up to 10 μM) were required to produce similar effects on current kinetics and the peak K^+^ current amplitudes remained unchanged (Fig. 6A, *right*). At the lower concentrations Aβ_1–42_ produced no significant effect on potassium currents (data not shown). Freshly prepared Aβ_1–42_ solutions (aggregated for less than 1 hr) had no effect on K^+^ current properties (Fig. 6A, *left*), whereas oligomers aggregated for 48 hrs facilitated current activation (Fig. 6B, C) and accelerated inactivation kinetics in a voltage-dependent manner (Fig. 6D, E). Since these preparations are entirely free of HFIP, these results show that the effects on Kv 1.3 current are specific for Aβ_1–42_ oligomers. For BLM recordings, we used Aβ_1–42_ at concentrations up to 2 µM. The increase of bilayer conductance was observed at concentrations as low as 0.25 µM Aβ_1–42_ (Fig. 6F, G). Overall, these results demonstrate that Aβ_1–42_ oligomers modulate Kv 1.3 potassium channel properties and lipid bilayer conductances. The potency of these effects depends on the oligomer preparation method, suggesting that fluorinated solvents, such as HFIP, may significantly alter properties of the resulting Aβ_1–42_ oligomer samples, augmenting their effects on membranes and ion channels.

## Discussion

We demonstrate in this study that Aβ_1–42_ oligomers, likely causal agents of AD, modulate the kinetics properties of the voltage-gated Kv 1.3 channels heterologously expressed in mammalian cells, and confirm that such aggregates also affect properties of artificial lipid bilayer membranes. In distinction from earlier work [28], we characterize the composition of the peptide aggregates here more rigorously with respect to residual fluorinated solvents and observe these effects regardless of whether the Aβ samples were prepared by dissolving the amyloid peptide in HFIP or in NaOH to initiate aggregate formation (Figures 4, 5, 6). This implies that residual fluorinated compounds, HFIP or TFA, in synthetic Aβ_1–42_ samples cannot account for the observed effects and shows that small soluble amyloid oligomers modulate voltage-gated potassium channels and interact with cell membranes, resulting in dysregulation of ionic homeostasis, which may ultimately lead to cell death [36].

It was previously shown that Aβ oligomers modulate voltage-gated calcium [5], [17], [20], [37] and potassium channels [16], [18], [19], [38], [39] directly or indirectly, by changing the properties of the membrane. It is unlikely, however, that the effects on Kv 1.3 current kinetics reported here are mediated by membrane-related effects because we did not observe any consistent changes in the resting current, cell capacitance or resistance in the presence of oligomers. The finding that Kv 1.3 channels are affected by amyloid oligomers opens the possibility that Kv 1.3, and potentially other *Shaker* family-related K^+^ channels, might be involved in the cascade of events leading to the dysfunction of synaptic transmission in the brain in AD. Indeed in the rodent brain, Kv 1.3 channels are expressed in the structures implicated in AD, such as the olfactory bulbs, olfactory cortex, hippocampus, and cerebellar cortex [40], [41], [42], [43], [44], [45], [46], where they have differential subcellular localization and regulate the repolarization of action potentials and neuronal firing patterns [47]. Speeding up of the activation and C-type inactivation kinetics of Kv 1.3 by Aβ oligomers may cause significant changes in neuronal firing rates and thus disrupt normal synaptic transmission. While the present work provides no direct evidence for binding of Aβ oligomers to Kv 1.3, it can not be excluded that synaptic accumulation of soluble Aβ oligomers, facilitated by increased synaptic activity [48], might be associated with modulation of presynaptic Kv channels by Aβ oligomers. In addition, modulation of Kv 1.3 properties by Aβ oligomers may also affect their non-traditional scaffolding functions [49], such as interaction with neurotrophic tyrosine receptor kinase B (trkB) [46], [50], [51] and β-integrins [52], [53]. Thus, it can not be excluded that Aβ effects on Kv 1.3 are contributing to impaired BDNF/trkB signaling in AD brain [53], [54], [55]. Furthermore, integrin-dependent activation of microglia by Aβ [56], [57] induces up-regulation of Kv 1.3 channels [58], [59] which, in turn, is crucial for maintenance of the activation state of microglia. This would suggest a link between Kv 1.3 modulation by Aβ and functional changes in microglia in the course of AD. Finally, Kv 1.3 channels have been linked to proliferation of neuronal progenitor cells [60]. Growing evidence suggests that neurogenesis is altered in AD patients [61], [62], [63], [64], [65], [66] and AD animal models [67], [68], [69], [70], and it is possible that modulation of Kv 1.3 channels by Aβ oligomers may contribute to these changes.

An equally important result is the observation that HFIP, a frequently used solvent in peptide chemistry, can be retained in peptide aggregate samples even after extensive evaporation. As Fig. 2(D, E) shows, ^19^F NMR provides a facile and sensitive means to quantify HFIP concentration in aqueous solution with or without Aβ_1–42._ We confirm that HFIP *per se* alters the kinetics properties of Kv 1.3 and, as we report here, increases the conductance of BLMs [29] as well. However, these effects only occur at HFIP concentrations that are much higher than those of the residual solvent detected in HFIP-prepared Aβ_1–42_ oligomer samples. The effects of HFIP alone on Kv 1.3 kinetics and BLM conductance (Fig. 3) can be compared with those of HFIP-containing Aβ_1–42_ aggregates prepared with *HFIP protocol I* (Fig. 4) where the HFIP concentration in Aβ_1–42_ stock solution with an amyloid concentration of 70 µM was 10.4±1.7 mM. Thus when diluted to 1 µM peptide, the HFIP concentration in the electrophysiology measurements is expected to be ≈140 µM. At this HFIP concentration, the current across a BLM under the conditions specified in Fig. 3 would be <10 pA. In the presence of Aβ_1–42_ oligomers, the current is >100 pA (see upper abscissa in Fig. 4H). Thus, residual HFIP alone cannot account for the increased lipid bilayer conductance induced by HFIP-prepared oligomers, even when the evaporation is performed with the less efficient separation procedure, *HFIP protocol* *I*. The same conclusion is reached by analyzing the effects of HFIP alone and of HFIP-prepared oligomers on the kinetics of Kv 1.3. Figure 3A shows the effects of 3 mM HFIP on the kinetics and amplitude of Kv 1.3 currents. Comparison of these results with the effects of 1 µM Aβ_1–42_ oligomers prepared according to *HFIP protocol* *I*, Fig. 4, shows that the estimated HFIP concentration in the samples (≈140 µM) is far too small to account for the large effect on Kv 1.3 peak currents and kinetics. The difference between the electrophysiological effects of HFIP-prepared oligomers from those of equivalent amounts of freely dissolved HFIP is even larger for aggregates prepared following *HFIP protocol* *II*. Here, the HFIP concentration in the peptide stock solution (≈100 µM Aβ_1–42_) is 1.7±1 µM. Thus, the estimated HFIP concentration in electrophysiology measurements at 1 µM Aβ_1–42_ is 0.017 µM. The intrinsic HFIP effects at this level are too small to observe above noise in BLM current measurements (note the nonlinear dose-response curve in Fig. 3G) or to distinguish from control in Kv 1.3 current measurements. Thus the effects observed with Aβ oligomers prepared by *HFIP protocol* *II* cannot be due to residual HFIP.

In the light of the hypothesis that endogenous Aβ oligomers affect neuronal cells by altering membrane properties or modulating voltage-gated ion channels, the critical question is whether or not HFIP-free amyloid aggregates, such as those prepared following the *NaOH protocol*, elicit measurable effects on membranes and channels. Figure 6 shows that Aβ_1–42_ oligomers prepared by *NaOH protocol* alter Kv 1.3 currents kinetics and increase the BLM conductance substantially at amyloid peptide concentrations of ≈10 µM. We thus conclude that amyloid oligomers themselves affect properties of Kv 1.3 potassium current and lipid bilayer membranes. On the other hand, our study determines clear quantitative differences between the effects of Aβ_1–42_ oligomers prepared by the different protocols, in particular between *HFIP protocol I* versus *HFIP protocol* *II* and *NaOH protocol*. While *protocol* *II* aggregates show similar characteristics to *NaOH protocol* aggregates, *HFIP protocol* *I* produces aggregates that are significantly more effective on membranes (compare Fig. 4 with Figs. 5 and 6). Because the concentration of HFIP in Aβ samples prepared by *HFIP protocol* *I* is too low to account for the observed effects by itself, these data suggest that HFIP interacts with Aβ peptide, potentially affecting intrinsic properties of the amyloid aggregates or the aggregation process.

In DLS, the scattered light intensity depends on the hydrodynamic radius of the particles as *I*(*d_h_*)∼*d_h_* ^6^. Therefore, in polydisperse samples the scattering from larger aggregates obliterates that from smaller ones. The reported size distributions (Fig. 1A–F) with main peaks around *d_h_*≈40 nm, ≈41 nm, and ≈21 nm for the *HFIP protocols* *I and II* and the *NaOH protocol* particles, respectively, do not rule out the presence of smaller Aβ_1–42_ aggregates in solution that would go undetected by DLS. It is, in fact, unlikely that the 20 nm peaks correspond to the smallest aggregate sizes present in the solutions. The number of peptide molecules contained in a spherical aggregate of the hydrodynamic diameter *d_h_*, which includes a hydrodynamically stagnant layer of solvent, can be roughly estimated as follows. With *V_h_*, *V_p_* and *d_p_* denoting, respectively, the hydrodynamic volume and the volume and diameter of the peptide core, the stagnant solvent layer occupies a volume, *V_h_*−*V_p_* = (π/6)(*d_h_* ^3^−*d_p_* ^3^). Accounting for the packing efficiency, φ, of peptides in an aggregate, the volume occupied by peptide in an aggregate is φ*V_p_*. Assuming that a stagnant layer around the dissolved aggregate is ≈10 Å thick [71], φ≈0.74 [72], and the average (hydrated) amino acid volume to be *v_aa_*≈ 200 Å^3^, an upper limit for the number of Aβ_1–42_ peptides in an aggregate is given by *n_p_*≈φ*V_p_*/(*v_aa_*×42)≈270 and *n_p_*≈2400 for samples prepared with *HFIP protocol* *II* and the *NaOH protocol*, respectively. From these estimates, it appears that the aggregates observed in DLS are not the oligomer species that are believed to be the origin of the biophysical effects on membranes and toxicological effects on neuronal cells [25], [26], [27]. Rather, it is likely that these samples contain smaller oligomers, invisible to DLS, which might be identified by their immunological signatures. The smaller proportion of the larger aggregates revealed by DLS in the samples prepared by *HFIP protocol* *I* compared to other protocols is associated with the strongest effect of Aβ on Kv 1.3 channels as well as on the BLM, suggesting that smaller aggregates are affecting properties of the Kv 1.3 channels and lipid bilayers in our experiments. Finally, the analysis of DLS data suggests that the effective concentrations of the oligomers of smaller sizes in Aβ samples would be much lower than total peptide concentrations in the Aβ samples.

Although the dot-blot analysis is insufficient to estimate the proportion of particular oligomeric species that react with the A11 or OC conformation-specific antibodies, the results in Fig. 1G demonstrate the presence of both A11- and OC-positive oligomers in Aβ samples regardless of the protocol used. Therefore, while aggregate populations or oligomer sizes determined by DLS and their effects on membranes and K^+^ current may differ in different preparations, the staining of all these preparations by OC and A11 antibodies implies that they contain oligomeric species with similar antigenic epitopes, indicating similar peptide conformations and supramolecular peptide aggregate structures. This implies again that all preparation protocols produce Aβ oligomers that affect Kv 1.3 currents and BLM conductivities and is consistent with the interpretation that peptide aggregates, not residual HFIP, is in large parts responsible for the effects observed with *protocol* *I* samples.

Overall, our data demonstrate that although the presence of trace amounts of HFIP in the synthetic Aβ samples cannot account for the effects on K^+^ current and membrane conductance, the synergistic interaction between HFIP and Aβ oligomers can not be excluded. Thus, much higher concentrations of Aβ are required to observe the effects on potassium channels and lipid bilayers when HFIP-untreated samples are tested. Alternatively, the properties of the aggregates formed with and without HFIP might differ. If, in fact, the higher potency of HFIP-prepared versus NaOH-prepared Aβ oligomers that we report is associated with HFIP effects on the aggregation process, the aggregation pathway of endogenous Aβ in the brain might also be affected by other fluorinated substances. Indeed, the interactions between Aβ and fluorinated inhalational anesthetics are well documented [73], [74], [75], [76]. HFIP is structurally related to sevoflurane and is a sevoflurane metabolite while TFA is an isoflurane metabolite. It is intriguing to speculate that interactions between fluorinated anesthetics and/or their metabolites with Aβ may affect the formation of aggregates in the brains of patients undergoing general anesthesia. Such interactions may underlie a previously proposed link between general anesthesia and Alzheimer's disease [77], [78], [79], [80].

### Conclusions

This study demonstrates that small Aβ_1–42_ aggregates affect the kinetics of voltage-gated Kv 1.3 channels in mammalian cells and increase membrane conductance. In HFIP-prepared Aβ peptide samples, however, these effects are more potent, possibly due to differences in the intrinsic properties of the oligomers. Because both HFIP-dissolved and HFIP-free Aβ samples produced qualitatively similar effects, preparing HFIP-free Aβ samples for testing amyloid effects in biological assays is preferable, as it eliminates any contribution from the solvent.

## Materials and Methods

### Preparation of Aβ oligomers

Aβ_1–42_ was synthesized as previously described [81]. Lyophilized peptides were resuspended in 50% acetonitrile in water and re-lyophilized. Several protocols, as described in detail below, were used to prepare solutions of Aβ_1–42_ oligomers.

#### HFIP protocol I: Standard Aβ oligomer preparation using HFIP as a solvent [28]

0.3 mg of Aβ_1–42_ were dissolved and incubated in 130 µL HFIP for 20 min in 1.5 mL siliconized Eppendorf tubes, followed by addition of 900 µL DD H_2_O and 20 min incubation in the resulting HFIP/water mixture. Subsequently, the samples were centrifuged at 14,000 g for 15 min. The solvent was evaporated from resulting supernatant under constant stirring at room temperature in two steps: (1) exposed to a gentle stream of argon for 10 min; (2) in ambient air with the Eppendorf tubes closed with perforated caps for 48 hr. The resulting Aβ_1–42_ concentration was typically 70 µM.

#### HFIP protocol II: Modified Aβ oligomer preparation using HFIP as a solvent

The protocol described above was modified in the last evaporation step where Eppendorf tubes were left open (no caps), allowing more efficient evaporation of the solvent. The resulting Aβ_1–42_ concentration in these samples was typically 100 µM.

#### NaOH protocol: Preparation of HFIP-free Aβ oligomer samples using NaOH as a solvent

0.3 mg Aβ_1–42_ were dissolved in 30 µL of 100 mM NaOH and incubated for 25 min, followed by the addition of 800 µL 10 mM sodium phosphate buffer or Ringer solution containing 10 mM HEPES. The resulting Aβ_1–42_ concentration was 70 µM. These samples were subsequently kept in closed Eppendorf tubes at room temperature. HEPES-buffered samples were used in the patch-clamp experiments to avoid any effect of phosphate buffer on recorded currents.

### Patch-clamp

The mouse fibroblast cell line L929 that stably expresses mouse Kv 1.3 potassium channel α subunits was maintained as previously described [82]. Patch-clamp experiments were performed in whole-cell mode using an EPC-9 (HEKA Elektronik) amplifier. Pipettes pulled from borosilicate glass capillaries (Garner Glass) using a Fleming/Brown micropipette puller (Sutter Instrument Co.) had resistances of 1.8–2.5 MΩ when filled with the internal recording solution that contained 145 mM KF, 10 mM EGTA, 10 mM HEPES, and 2 mM MgCl_2_ (pH 7.3). The ground electrode was connected to the bath via an agar bridge. The external solution contained 2 mM CaCl_2_, 1 mM MgCl_2_, 4.5 mM KCl, 155 mM NaCl, 10 mM D-glucose, and 5 mM HEPES. Outward K^+^ currents were evoked in voltage-clamp mode by 400 ms long depolarizing voltage pulses from −80 mV holding potential to +40 mV with 10 mV increments and 30 s inter-pulse intervals to prevent the cumulative inactivation characteristic of Kv 1.3. The sampling frequency was 10 kHz. The liquid junction potential was corrected during data acquisition. The cell and pipette capacitances were compensated during recordings; leak currents were not subtracted. Data files were recorded using PULSE/PULSEFIT (HEKA Elektronik) and analyzed using OriginPro7.5. For the analysis of I_K+_ activation kinetics, the currents were normalized to their peak values and the data were fitted with sigmoidal functions to determine activation time constants. The inactivation time constants were determined by fitting the data to single-exponential decay function.

To test the effects of HFIP and TFA, fresh stocks in the External solution were prepared before each experiment and needed concentrations were added directly to the gravity-driven perfusion system. To test the effects of Aβ_1–42_ peptide, the needed amounts of stock solution were added directly to perfusion system prior to testing the effects. Although all the effects were not time-dependent and were observed as soon as the drugs reached the cells, the cells were exposed to each concentration for 8–10 minutes for complete solution exchange in the recording chamber. To ensure that the effects are not due to changes in the current or cell parameters, the application of the drugs was done 15–20 min after seal formation and only when cell parameters as well as the current is stabilized so that at least three consequent control recordings completely overlapped and the cell parameters are not changed.

### Lipid bilayer conductance

Dioleoylphosphatidylcholine (DOPC) and dioleoylphosphatidylethanolamine (DOPE) (Avanti Polar Lipids) were mixed 1∶1 to form BLMs. Electrolyte solutions of various KCl or NaCl concentrations were buffered with 1 or 10 mM HEPES-Tris at pH 7.4. BLMs were formed across an aperture (diameter ≈150 µm) in a 15 µm thick Teflon (PTFE) septum, punched by an electric spark and precoated with 2.5% squalane in *n*-pentane, by raising the buffer levels underneath two phospholipid monolayers of the appropriate phospholipid mixtures separated by the septum [27]. Bilayer formation was monitored by measuring conductance with silver/silver chloride wires used as electrodes to apply voltages and record currents. The rear chamber potential was taken as ground and additions were made to the front chamber where the solutions were stirred with magnetic stirrer bars for at least 30 s after each addition. For measurements of membrane conductance, a voltage ramp protocol (−150 to +150 mV, at 60 mV/s) was used. All experiments were performed at room temperature. Voltages were generated and currents digitized at a resolution of 12 bits with JCLAMP (SciSoft Co.) driving a National Instruments NI PCI 6024E board. Currents were transduced by an Axopatch 200 A amplifier (Axon Instruments) connected to the National Instruments board through an NI BNC2090 interface panel.

### Dynamic light scattering

DLS experiments were performed using a Malvern Zetananosizer (Malvern, UK) at a scattering angle of 173° in a correlator time lag window of 0.5 µs–1 s. Distributions of diffusion constants for Aβ_1–42_ aggregates were obtained through the regularized inverse Laplace transform (RILT) of experimental correlograms. Size distributions were then obtained assuming a Stokes-Einstein relation, *D = k_Β_T/*(3πη*d_h_*), between the diffusion constant *D* and the aggregate hydrodynamic diameter, *d_h_*.

### ^19^F NMR spectroscopy

^19^F NMR spectra were recorded at 298 K using a Bruker DRX400 spectrometer at 376 MHz and a 5 mm QNP (^1^H/^13^C/^31^P/^19^F) NMR probe. *XwinNMR* (Bruker) was used for data acquisition and processing. A separate CFCl_3_ sample was used as a reference standard for reporting the ^19^F chemical shifts. All samples contained 3-(trimethylsilyl)-1-propanesulfonic acid sodium salt, used to manually shim the spectrometer by observation of the proton signal. A concentration standard was generated from HFIP- and TFA-spiked samples without peptide to quantify concentrations of residual fluorinated substances in peptide samples.

### Dot blot immunochemistry

One μL of each sample was applied to a nitrocellulose membrane that had been blocked with 10% non-fat milk in Tris-buffered saline (TBS) containing 0.05% Tween 20 (TBS-T) at room temperature for 1 h, washed three times for 5 min each with TBS-T and incubated overnight at 4°C with the conformation-specific antibodies OC or A11 [7], [83] in 5% milk/TBS-T. Subsequently, the membranes were washed three times for 5 min each with TBS-T, and incubated with horseradish peroxidase-conjugated anti-rabbit IgG (Promega) diluted 1∶10,000 in 5% milk/TBS-T for 1 hour at room temperature. The blots were washed again three times with TBS-T, one time with TBS, and then developed with SuperSignal West Femto Maximum Sensitivity Substrate kit from ThermoScientific (Rockford, IL).

### Statistical analysis

The results are presented as mean ± S.E.M. For electrophysiological experiments statistical differences were determined by two-way Repeated Measures (RM) ANOVA and Student's t-Test for two-group comparisons. In order to confirm that each data set is a normally distributed population of observations, we used the Shapiro-Wilk normality test for each data set prior to performing paired t-Test. Analyses were performed using OriginPro version 8 (OriginLab Corporation, Northampton, MA 01060).
